# Packing Vulcanite

**Published:** 1919-02

**Authors:** W. M. Randall


### PACKING VULCANITE

BY W. M. RANDALL, D.D.S.

If we are going to make the very best dentures in vulcanite, there is a type of technic that I personally believe is important to us. We all recognize the strength of plaster, and plaster is more or less affected by the egress of moisture, and still the majority of us insist that we should boil our plaster to remove the wax, and boil it and heat it and press up our vulcanite; and what are we doing? We put it in the vulcanizer and vulcanize it, and immerse it in water, all the while saturating that plaster, and weakening it the more it becomes saturated.

For several years it has been recognized that plaster becomes stronger and stands more pressure as it becomes dried out; and I have used what is called the dry heat technic, a method of dry heat, using Wilmot's sterilizer. There are different methods of doing it—where you can shut off the steam from the water and have a chamber of dry heat. Put the flask in it and leave it at a certain temperature, which is registered by the thermometer; and when it reaches a certain point, the wax can be removed en masse and the flask is separated, and only the minimum quantity of boiling water—with tin foil next to the plaster—will remove all the wax from the pins. I then use dry heat in warming the plaster previous to packing, and get the plaster thoroughly dry. And when you pack the vulcanite against the warm moulds of plaster, it will lie just where it is put, and will remain there. You can more accurately pack. There is less excess of vulcanite. The plaster is getting harder all the while.

I prefer a vulcanizer which will keep the flask out of the water, free from contact with anything other than superheated steam above the surface of the water. In getting anything like an accurate registration by the thermometer, all the air should be expelled from the vulcanizer, and in vulcanizing the rubber should be out of the water, and with dry plaster in the flask, pressing it down and closing the flask completely, not at 212 but at 248, which is recognized as the fusing point or the most liquid state of vulcanite.

With this technic, with that maximum of the plaster in the flask, we have the minimum resistance offered by the vulcanite; we have the most perfect adaptation that can be obtained in the closing of the flasks.

The result is a less risk in change of the position of the teeth, the articulation of which is carried out so accurately, as was shown yesterday; and we do not want to run any risk.

I would suggest that you try this type of technic in carrying out the rubber cases. I am sure it will be most satisfactory to you in most cases, used in conjunction with the tin foil on both sides of the vulcanite. There is a great deal in the strength of vulcanite, in its texture and in its elasticity, in the process of vulcanizing, which I could not attempt to describe at this time.—*Journal N. D. A.*

